# Deprescribing Antipsychotics Based on Real-World Evidence to Inform Clinical Practice: Safety Considerations in Managing Older Adults with Dementia

**DOI:** 10.3389/fphar.2021.706750

**Published:** 2021-11-26

**Authors:** Stephanie Hsieh, Jing Yuan, Z. Kevin Lu, Minghui Li

**Affiliations:** ^1^ Department of Pharmacy, Scarborough Health Network – Centenary Hospital, Scarborough, ON, Canada; ^2^ Department of Clinical Pharmacy and Pharmacy Administration, Fudan University, Shanghai, China; ^3^ Department of Clinical Pharmacy and Outcomes Sciences, University of South Carolina, Columbia, SC, United States; ^4^ Department of Clinical Pharmacy and Translational Science, University of Tennessee Health Science Center, Memphis, TN, United States

**Keywords:** dementia, antipsychotics, deprescribing, real-world evidence, older adults

## Abstract

**Background:** Antipsychotics are commonly used in dementia patients but have potential risks that often outweigh clinical benefits. Limited studies have assessed the healthcare utilization and medical costs associated with antipsychotic use, especially those focused on cumulative days of use.

**Objectives:** To examine clinical and economic burdens associated with different cumulative days of antipsychotic use in older adults with dementia in the United States.

**Methods:** This study used Medicare Current Beneficiary Survey (2015–2017). Older (≥65 years) Medicare beneficiaries with dementia, without concurrent schizophrenia, bipolar disorder, Huntingon’s disease, or Tourette’s syndrome were included. Antipsychotic use was measured using Medicare Part D prescription events. Healthcare utilization was measured as inpatient services, outpatient services, and emergency room (ER) visits. Total medical costs were classified as Medicare and out-of-pocket costs. The logistic regression, negative binomial regression, and generalized linear model with a log link and gamma distribution were used to examine factors, healthcare utilization, and medical costs. Survey sampling weights were applied to generate national estimates.

**Results:** Among older adults with dementia, 13.18% used antipsychotics. Factors associated with antipsychotic use were being Hispanic (OR: 2.90; 95% CI: 1.45, 5.78), widowed (OR: 3.52; 95% CI: 1.46, 8.48), and single (OR: 3.25; 95% CI: 1.53, 6.87). Compared to non-users, antipsychotic use was associated with higher inpatient visits (IRR: 2.11; 95% CI 1.53, 2.90), ER visits (IRR: 1.61; 95% CI: 1.21, 2.13), total costs (β: 0.53; 95% CI: 0.36, 0.71), Medicare costs (β: 0.49; 95% CI 0.26, 0.72), and out-of-pocket costs (β: 0.66; 95% CI: 0.35, 0.97). With the increase in cumulative days of antipsychotic use, the magnitude of clinical and economic burdens was decreased.

**Conclusion:** The significant clinical and economic burdens associated with antipsychotic use, especially with short-term use, provide real-world evidence to inform clinical practice on deprescribing antipsychotics among community-dwelling geriatric dementia patients.

## Introduction

Dementia is a syndrome characterized by progressive cognitive function deterioration with various etiology (van der Flier and Scheltens, 2005). Alzheimer’s disease is the most common type of dementia, which is estimated to affect more than five million older adults aged 65 years and over (Alzheimer’s Association, 2020). The public health impact of dementia is significant. Individuals with dementia utilized hospital and emergency room (ER) services two times more frequently than those without dementia (Alzheimer’s Association, 2020). The total healthcare costs incurred by individuals with Alzheimer’s disease are estimated to be more than $300 billion and the indirect costs (e.g., care provided by unpaid caregivers) are estimated to value more than $200 billion in the United States (Alzheimer’s Association, 2020).

One of the most stressful and costly aspects of dementia care involves the management of behavioral and psychological symptoms of dementia (BPSD) (Kales et al., 2015). BPSD encompasses a range of symptoms caused by disturbances in the individual’s mood, behavior, thoughts, and perception; symptoms of BPSD include agitation, psychosis, and aggression (Kales et al., 2015). It is highly prevalent and is estimated to affect up to 80% of individuals with dementia (Kirkham et al., 2017). Non-pharmacological treatment is recommended as first-line for its management to reduce the risks of adverse events with pharmacological therapies (Reus et al., 2016; Kirkham et al., 2017). Family caregiver interventions, environmental strategies, and patient-oriented approaches are all potential non-pharmacological approaches that can be attempted initially to manage BPSD (Kales et al., 2015). However, these strategies might not always be effective and appropriate when BPSD symptoms are severe, dangerous, and cause significant patient distress (Reus et al., 2016).

When non-pharmacological therapy fails or is not appropriate alone for BPSD symptoms, clinical practice guidelines suggest that pharmacological therapy might be used (Reus et al., 2016). Among various therapeutic options, antipsychotics are the most extensively studied and commonly used agents (Liperoti et al., 2008; Kales et al., 2015; Kirkham et al., 2017). Antipsychotic agents block D2 receptors in various cerebral regions to modulate the effects of dopamine and, subsequently, BPSD symptoms (Liperoti et al., 2008).

The use of antipsychotics in older adults with dementia requires a careful assessment of clinical benefit for BPSD symptoms against their potential risks (Reus et al., 2016). Serious adverse events, such as mortality and cerebrovascular accidents, are found to be associated with antipsychotic use in older adults (Maust et al., 2015; Tampi et al., 2016; Zhai et al., 2016). The U.S. Food and Drug Administration has issued a black box warning advising the increased mortality risk associated with antipsychotic use in patients with dementia (Kim et al., 2011). Some medical societies have advised the judicious use of antipsychotics in treating BPSD symptoms in dementia, including American Geriatrics Society through its Beers Criteria, American Board of Internal Medicine through their Choosing Wisely campaign, and the American Psychiatric Association (Reus et al., 2016; American Geriatrics Society Beers Criteria® Update Expert Panel, 2019). However, the use of antipsychotics remain common in dementia patients (Kirkham et al., 2017).

Limited studies have assessed the healthcare utilization and medical costs associated with antipsychotic use, especially those focused on cumulative days of use. Evidence on whether antipsychotic use among older adults with dementia was associated with an increase in acute hospital admissions is conflicting. (Raivio et al., 2007; Rochon et al., 2008). Older adults with Alzheimer’s disease were more likely to visit the emergency department due to psychotropic-related adverse drug effects, with antipsychotic agents commonly implicated in these visits (Sepassi and Watanabe, 2019). Total healthcare costs were significantly greater among patients with Alzheimer’s disease who used second-generation antipsychotics (Rosenheck et al., 2007). The clinical utility of existing studies is limited as most did not examine cumulative days of antipsychotic use and did not differentiate between different types of healthcare utilization and medical costs. To fill the gap in the literature, the objectives of this study were: 1) to evaluate factors associated with antipsychotic use, 2) to examine healthcare utilization associated with cumulative days of antipsychotic use, and 3) to assess medical costs of cumulative days of antipsychotic use in older adults with dementia.

## Methods

### Data Source

This study used the Medicare Current Beneficiary Survey (MCBS) from 2015 to 2017. The MCBS is a nationally representative survey of Medicare beneficiaries conducted by the Centers for Medicare and Medicaid Services in the United States (Medicare Current Beneficiary Survey (MCBS), 2019). Through linking Medicare administrative, claims, and survey data, the MCBS collects comprehensive information on demographic and socioeconomic characteristics, health status, healthcare utilization, and medical costs from Medicare beneficiaries.

### Study Population

This study included Medicare beneficiaries who were 65 years of age and over, lived in the community setting, had a diagnosis of dementia or had two or more dementia prescriptions with more than 60-days supply, and had continuous coverage of Medicare Part A, B, and D. Medicare beneficiaries who were enrolled in the health maintenance organization and had a diagnosis of schizophrenia, bipolar disorder, Huntingon’s disease, or Tourette’s syndrome were excluded.

### Measurement

Antipsychotic use was measured based on Medicare Part D prescription events, as defined by the Pharmacy Quality Alliance (PQA). Antipsychotics measured in this study included aripiprazole, asenapine, brexpiprazole, cariprazine, chlorpromazine, clozapine, fluphenazine, haloperidol, iloperidone, loxapine, lurasidone, molindone, olanzapine, paliperidone, perphenazine, pimavanserin, pimozide, quetiapine, risperidone, thioridazine, thiothixene, trifluoperazine, and ziprasidone. Cumulative days of antipsychotic use were calculated as the total number of days of antipsychotic use in a measurement year. Dementia was measured using the relevant 10th revision of the International Statistical Classification of Diseases and Related Health Problems (ICD-10) codes from Medicare Part A and B claims. Healthcare utilization was measured as inpatient services, outpatient services, and emergency room (ER) visits and was collected from Medicare Part A and B claims. Total medical costs were collected from Medicare claims and self-reports. Total costs were further classified as Medicare and out-of-pocket (OOP) costs based on different payers. Medical costs in different years were adjusted to 2017 dollars using the consumer price index of medical care services. Covariates considered in this study included age, gender, race/ethnicity, education, marital status, income, residence, census region, and comorbidity.

### Statistical Analyses

Characteristics of the study population were compared between users and non-users of antipsychotics by the Chi-square test. The t-test was used to compare healthcare utilization and medical costs between users and non-users of antipsychotics. The logistic regression model was used to identify factors associated with antipsychotic use. Healthcare utilization was analyzed using the negative binomial regression for count data. Medical costs were analyzed using the generalized linear model with a log link and gamma distribution. Survey sampling weights were applied to generate national estimates. All analyses were performed using SAS software, version 9.4 (SAS Institute, Inc., Cary, NC).

## Results

After applying the inclusion and exclusion criteria, 4,953,945 (weighted number) older adults with dementia were included in this study. The majority of respondents were at least 85 years old (44.71%), female (66.74%), non-Hispanic whites (78.66%), high school graduate (32.93%), widowed (46.55%), had an annual household income between $10,000 and $19,999 (30.95%), lived in metropolitan areas (77.59%), lived in the South (41.76%), and had a Charlson comorbidity index (CCI) of three and higher (41.61%). (Table 1). Among older adults with dementia, 13.18% (weighted number = 652,799) used antipsychotics. Compared to those who did not use antipsychotics, antipsychotic users were more likely to be racial and ethnic minorities (*p* = 0.0305) and not married (*p* = 0.0249). (Table 1). Among users of antipsychotics, 16.51% used 1–30 days, 13.39% used 31–90 days, 19.04% used 91–180 days, 14.29% used 181–300 days, and 36.76% used more than 300 days.

**TABLE 1:** Characteristics of older adults with dementia and their use of antipsychotics (Weighted *n* = 4,953,945).

	Total	Antipsychotic use		
	*n* = 4,953,945	No	Yes	*p*
		*n* = 4,301,145	*n* = 652,799	
—	%	%	%	
Age	—	—	—	0.7871
65–74	18.65	18.27	21.14	—
75–84	36.64	36.80	35.61	—
85+	44.71	44.93	43.25	—
Gender	—	—	—	0.9623
Female	66.74	66.78	66.50	—
Male	33.26	33.22	33.50	—
Race/ethnicity	—	—	—	0.0305
Non-Hispanic white	78.66	79.76	71.43	—
Non-Hispanic black	9.67	8.91	14.70	—
Hispanic	7.22	6.59	11.40	—
Other	4.45	4.75	2.47	—
Education	—	—	—	0.7191
Less than high school	25.72	25.92	24.28	—
High school graduate	32.93	32.93	32.96	—
Some college	19.14	19.66	15.49	—
College graduate	22.21	21.50	27.27	—
Marital status	—	—	—	0.0249
Married	37.78	39.62	25.60	—
Widowed	46.55	45.53	53.36	—
Single	15.67	14.86	21.04	—
Income	—	—	—	0.8951
<$10,000 per year	17.09	16.81	18.94	—
$10,000–19,999 per year	30.95	31.31	28.57	—
$20,000–39,999 per year	23.79	23.85	23.39	—
≥ $40,000 per year	28.17	28.03	29.11	—
Residence	—	—	—	0.1354
Metropolitan	77.59	76.81	82.74	—
Non-metropolitan	22.41	23.19	17.26	—
Census region	—	—	—	0.4381
Northeast	20.14	20.64	16.81	—
Midwest	24.05	24.61	20.34	—
South	41.76	40.76	48.40	—
West	14.05	13.99	14.45	—
CCI	—	—	—	0.4468
0	22.50	22.32	23.70	—
1	21.32	20.64	25.84	—
2	14.56	14.53	14.81	—
3+	41.61	42.52	35.65	—
—	Mean ± SD	Mean ± SD	Mean ± SD	*p*
Healthcare utilization				
Inpatient	0.58 ± 0.04	0.53 ± 0.04	0.91 ± 0.11	0.0011
Outpatient	6.72 ± 0.34	6.90 ± 0.36	5.55 ± 0.53	0.0189
ER admission	1.25 ± 0.07	1.17 ± 0.07	1.77 ± 0.19	0.0016
Medical costs ($)				
Total costs	53,336 ± 2,050	50,299 ± 2,172	73,345 ± 4,793	<0.0001
Medicare costs	23,053 ± 1,258	22,000 ± 1,378	29,990 ± 2,808	0.0100
OOP costs	14,942 ± 947	13,902 ± 966	21,792 ± 2,656	0.0057

CCI, charlson comorbidity index; ER, emergency room; OOP, out-of-pocket; SD, standard deviation.

Ethnicity and marital status were two characteristics associated with antipsychotic use in older adults with dementia identified in this study. Compared to non-Hispanic whites, Hispanics were significantly more likely to use antipsychotics [OR (odds ratio): 2.90; 95% CI (confidence interval): 1.45, 5.78] (Table 2). Widowed (OR: 3.52; 95% CI: 1.46, 8.48) and single older adults (OR: 3.25; 95% CI: 1.53, 6.87) were significantly more likely to use antipsychotics, compared to those who were married. (Table 2). Age, gender, education level, annual income, residence, census region, and comorbidity were not found to be associated with antipsychotic use.

**TABLE 2:** Factors associated with antipsychotic use in older adults with dementia.

	OR	95% CI
Age		
65–74	Ref	—
75–84	0.81	(0.37, 1.78)
85+	0.57	(0.26, 1.27)
Gender		
Female	Ref	—
Male	1.20	(0.65, 2.23)
Race/ethnicity		
Non-Hispanic white	Ref	—
Non-Hispanic black	1.87	(0.84, 4.20)
Hispanic	2.90	(1.45, 5.78)
Other	0.71	(0.24, 2.10)
Education		
Less than high school	Ref	—
High school graduate	1.42	(0.68, 2.99)
Some college	0.89	(0.40, 1.99)
College graduate	1.75	(0.70, 4.37)
Marital status		
Married	Ref	—
Widowed	3.52	(1.46, 8.48)
Single	3.25	(1.53, 6.87)
Income		
<$10,000 per year	Ref	—
$10,000–19,999 per year	1.12	(0.59, 2.12)
$20,000–39,999 per year	1.62	(0.84, 3.10)
≥ $40,000 per year	2.08	(0.98, 4.41)
Residence		
Metropolitan	Ref	—
Non-metropolitan	0.56	(0.30, 1.08)
Census region		
Northeast	Ref	—
Midwest	1.18	(0.48, 2.87)
South	1.91	(0.90, 4.03)
West	1.48	(0.67, 3.28)
CCI		
0	Ref	—
1	1.32	(0.65, 2.70)
2	0.78	(0.39, 1.58)
3+	0.77	(0.43, 1.38)

CCI, charlson comorbidity index; OR, odds ratio; CI, confidence interval.

**FIGURE 1:**
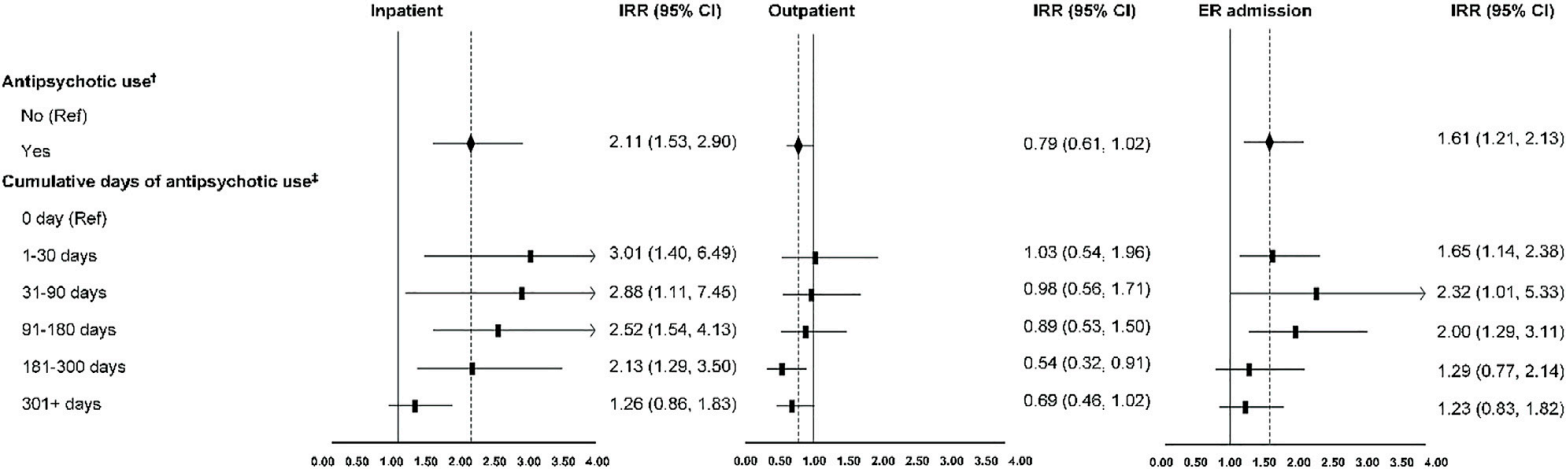
Healthcare utilization associated with antipsychotic use in older adults with dementia. ER indicates emergency room; IRR, incidence rate ratio; CI, confidence interval. ^†, ‡^: Covariates adjusted (age, gender, race/ethnicity, education, marital status, income, residence, census region, and comorbidity).

The annual number of inpatient visits was 0.58 (SD = 0.04), outpatient visits were 6.72 (SD = 0.34), and ER visits were 1.25 (SD = 0.07) on average among older adults with dementia. (Table 1). After controlling for covariates, antipsychotic use was associated with significantly more inpatient visits (IRR [incidence rate ratio]: 2.11; 95% CI: 1.53, 2.90) and more ER visits (IRR: 1.61; 95% CI 1.21, 2.13). (Figure 1). The magnitude of the association between antipsychotic use and healthcare utilization was decreased with the increase in cumulative days of antipsychotic use (Figure 1).

**FIGURE 2:**
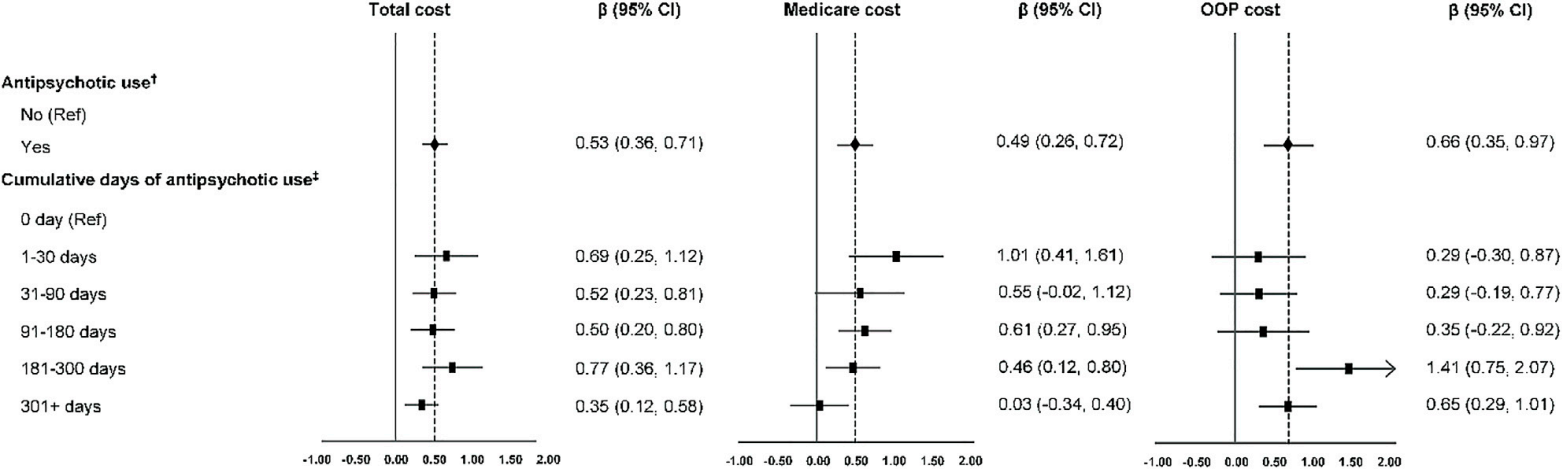
Medical costs associated with antipsychotic use in older adults with dementia. OOP indicates out-of-pocket; CI, confidence interval. ^†, ‡^: Covariates adjusted (age, gender, race/ethnicity, education, marital status, income, residence, census region, and comorbidity).

The annual total costs were $53,336 (SD = $2,050), Medicare costs were $23,053 (SD = $1,258), and OOP cost were $14,942 (SD = $947) on average among older adults with dementia (Table 1). After controlling for covariates, antipsychotic use was associated with significantly higher total costs (β: 0.53; 95% CI: 0.36, 0.71), higher Medicare costs (β: 0.49; 95% CI 0.26, 0.72), and higher OOP costs (β: 0.66; 95% CI: 0.35, 0.97) (Figure 2). The magnitude of the association between antipsychotic use and total and Medicare costs was decreased with the increase in cumulative days of antipsychotic use. However, the magnitude of the association between antipsychotic use and OOP costs was increased with the increase in cumulative days of antipsychotic use (Figure 2).

## Discussion

This study identified ethnicity as a factor associated with antipsychotic use among older adults with dementia. Specifically, we found that Hispanics were three-fold more likely to use antipsychotic agents compared to non-Hispanic whites. This is consistent with Xiong et al.’s study which found that Hispanics were 1.4-times more likely to take antipsychotics compared to non-Hispanic whites (Xiong et al., 2015). This ethnic disparity could be explained by greater dementia severity, more prevalent BPSD symptoms, language barrier, and cultural norm (Ferguson and Candib, 2002; Xiong et al., 2015). Xiong et al. found that Hispanics were more likely to have a higher severity of dementia, as well as a higher prevalence of BPSD symptoms across all dementia severity (Xiong et al., 2015). Additionally, the language barrier might limit patient-provider communication among Hispanic patients. Ferguson and Candib found that minority patients not proficient in English were more likely to have the quality of patient-provider communication and relationship adversely affected as the language barrier might prevent the establishment of rapport, the receipt of sufficient information, and patient participation in medical decision-making (Ferguson and Candib, 2002). For example, clinicians might have difficulties when communicating with Spanish-speaking patients to identify potentially inappropriate use of antipsychotics and thus might be less likely to engage in deprescribing activities. Furthermore, Spanish-speaking patients and/or caregivers might not be able to understand the risk-benefit information communicated to them, with regards to the use of antipsychotics in BPSD. The cultural norm of self-prescription could explain the higher likelihood of antipsychotic use in Hispanics with dementia (Coffman et al., 2008). As Central and South America allow the unrestricted sale of medications that would otherwise be strictly regulated in the U.S., self-prescribing and use of medications among resident Hispanics for self-care are highly prevalent (Coffman et al., 2008). Therefore, Hispanics with dementia might be less willing to try non-pharmacological therapies as they find more comfort in using antipsychotics or perceive antipsychotics as being more effective.

In addition to being of Hispanic ethnicity, this study found that being widowed and single were associated with a higher likelihood of using antipsychotics, compared to those who were married. Differences in marital status in antipsychotic use in older adults with dementia can be explained by the type of caregiver providing dementia care. The main caregiver of a married individual is usually the spouse. For a widowed or single individual, the main caregiver can be a child, a family member, a friend, or a nurse. Previous studies found differences in care provided by adult-child and spousal caregivers (Reed et al., 2014; Rigby et al., 2019). Reed et al. reported that adult-child caregivers providing care to patients with Alzheimer’s disease, spent less overall caregiving time (e.g., assisting with basic activities of daily living and supervising patients) compared to spousal caregivers (Reed et al., 2014). Rigby et al. reported that adult-child caregivers who provided care to patients with Lewy body dementia saw them less often compared to spousal caregivers (Rigby et al., 2019). Due to the time and effort involved in non-pharmacological treatments, a caregiver other than the spouse might be more willing to consider pharmacological treatments on dementia patients with behavior issues.

The use of antipsychotics was found to be associated with more inpatient and ER visits. Adverse events, especially serious ones, associated with antipsychotic use in patients with dementia might contribute to the increased inpatients and ER visits identified in this study. Rochon *et al.* found that antipsychotic use was associated with a higher likelihood of developing serious adverse events (defined as hospitalization or death) within 30 days of use, among community-dwelling older adults with dementia (Rochon et al., 2008). Serious adverse events contributing to the acute care hospital admissions within 30 days of antipsychotic use included fall or hip fracture, cerebrovascular event, extrapyramidal symptoms, and other adverse events (Rochon et al., 2008). This study found a decreasing trend in healthcare utilization with the increase in cumulative days of antipsychotic use. Previous studies found that the risk of serious adverse events associated with antipsychotics was the highest within the first few weeks of use (Ballard et al., 2011; Kales et al., 2015). Kales et al. reported that the mortality risk was 1.5-fold higher within the first 120 days of antipsychotic use and decreased in the following 60 days among older adults with dementia (Kales et al., 2015). The increased cerebrovascular adverse events were only significant within the first week of antipsychotic use but not significant over a longer duration of use, among older antipsychotic users (Ballard et al., 2011). This association might explain the higher healthcare utilization within the first 30 days of antipsychotic use found in this study.

This study also found that the use of antipsychotics among older adults with dementia was associated with a higher economic burden. This is consistent with the cost-benefit analysis of a clinical trial evaluating the effectiveness of second-generation antipsychotics in treating BPSD symptoms among outpatients with Alzheimer’s disease. (Rosenheck et al., 2007). Total healthcare costs were found to be significantly higher with the users of second-generation antipsychotics, compared to non-users. (Rosenheck et al., 2007). Our study further included older adults with other types of dementia and measured costs from different payers. With cumulative days of use, our study observed the highest total and Medicare costs within the first 30-days of use, which then decreased with continued use of antipsychotics. To our knowledge, this is a novel finding in the literature, since no existing studies have evaluated the effects of cumulative antipsychotic use on medical costs. The higher economic burden within the first 30 days could be related to the higher rates of mortality and cerebrovascular events in the short term and its associated healthcare utilization (Ballard et al., 2011).

The significant increase in clinical and economic burden associated with antipsychotic use observed in this study provides real-world evidence to support deprescribing these agents in older adults with dementia. Deprescribing refers to the planned discontinuation or dose reduction, under medical supervision, of a medication when the benefits of continued use or at the current dose no longer outweigh the risks (Bjerre et al., 2018). Based on our findings, deprescribing efforts might be most beneficial when performed with short-term use of antipsychotics. This is consistent with the recommendation from the 2016 American Psychiatric Association Practice Guideline on the use of antipsychotics to treat BPSD, which suggests that antipsychotics should be tapered and discontinued if patients have no clinically significant response after a four-week trial (Reus et al., 2016). Additionally, consideration of deprescribing is also recommended in patients who experience clinically significant adverse events any time after use and/or in those who have adequately responded to treatment after 4 months of use (Reus et al., 2016). The guidelines also highlight the importance of engaging patients and/or their caregivers when making clinical decisions regarding using and deprescribing antipsychotic agents (Reus et al., 2016). Based on the results of existing discontinuation studies, tapering and stopping antipsychotics can be done safely without symptom recurrence in many patients (Ballard et al., 2009; Reus et al., 2016). However, monthly or more frequent reassessment of patients undergoing tapering up to 4 months following successful discontinuation of these agents, is prudent to monitor for recurrence of BPSD symptoms (Reus et al., 2016).

As our study has highlighted that individuals of Hispanic ethnicity and those who are single or widowed are more likely to use antipsychotic agents, increased attention should be paid when prescribing antipsychotics to these patients. For example, healthcare providers should address language barriers by ensuring the availability of professional interpreters at medical visits for Hispanic patients who speak limited English and collaborate with caregivers to implement non-pharmacological interventions prior to initiating antipsychotic therapy when feasible. Furthermore, these individuals might be targets for initial deprescribing efforts. As the finding from our study suggests that successful discontinuation of antipsychotics among older adults with dementia might not only reduce adverse events associated with antipsychotic use, but might also reduce clinical and economic burden.

This study has some strengths and limitations. To our knowledge, this is the first study conducted in the U.S. assessing the impact of antipsychotic use on healthcare utilization and costs among older adults with dementia. This study further examined cumulative days of antipsychotic use to support clinical practice in deprescribing efforts. Among the limitations, this study may not have identified all dementia patients since dementia can be under-diagnosed or undiagnosed. Therefore, those who have not seeked care would not be captured in Medicare claims. In addition, the stage and severity of dementia and BPSD cannot be measured based on ICD-10 codes. Second, different types of antipsychotics were not examined in this study, and the dispensed prescription was used as a proxy for medication use. Third, this study was conducted on residents in the community rather than facility settings. Finally, indirect costs of antipsychotic use (e.g., productivity loss of family members caring for patients) were not assessed.

## Conclusion

Antipsychotic agents should be used judiciously in older adults with dementia due to the increased clinical and economic burden. Factors associated with antipsychotic use included being of Hispanic ethnicity and being of a widowed or single status. Healthcare utilization and medical costs were significantly increased with antipsychotic use. With the increase in cumulative days of antipsychotic use, the magnitude of clinical and economic burden was decreased. The significant clinical and economic burdens associated with users of antipsychotics, especially short-term users, provide real-world evidence to inform clinical practice on deprescribing antipsychotics among community-dwelling geriatric dementia patients.

## Data Availability

The data analyzed in this study is subject to the following licenses/restrictions: The data will not be made readily available for the privacy of the participants. Requests to access these datasets should be directed to: https://www.cms.gov/Research-Statistics-Data-and-Systems/Research/MCBS; MCBS@cms.hhs.gov.

## References

[B1] Alzheimer’s Association (2020). Alzheimer’s Disease Facts and Figures. Alzheimers Dement J. Alzheimers Assoc. 16 (3), 391–460. 10.1002/alz.12068

[B2] American Geriatrics Society Beers Criteria® Update Expert Panel (2019). American Geriatrics Society 2019 Updated AGS Beers Criteria® for Potentially Inappropriate Medication Use in Older Adults. J. Am. Geriatr. Soc. 67 (4), 674–694. 10.1111/jgs.15767 30693946

[B3] Ballard C. Creese B. Corbett A. Aarsland D. (2011). Atypical Antipsychotics for the Treatment of Behavioral and Psychological Symptoms in Dementia, with a Particular Focus on Longer Term Outcomes and Mortality. Expert Opin. Drug Saf. 10 (1), 35–43. 10.1517/14740338.2010.506711 20684745

[B4] Ballard C. Hanney M. L. Theodoulou M. Douglas S. McShane R. Kossakowski K. (2009). The Dementia Antipsychotic Withdrawal Trial (DART-AD): Long-Term Follow-Up of a Randomised Placebo-Controlled Trial. Lancet Neurol. 8 (2), 151–157. 10.1016/S1474-4422(08)70295-3 19138567

[B5] Bjerre L. M. Farrell B. Hogel M. Graham L. Lemay G. McCarthy L. (2018). Deprescribing Antipsychotics for Behavioural and Psychological Symptoms of Dementia and Insomnia: Evidence-Based Clinical Practice Guideline. Can. Fam. Physician 64 (1), 17–27. 29358245 PMC5962971

[B6] Coffman M. J. Shobe M. A. O'Connell B. (2008). Self-Prescription Practices in Recent Latino Immigrants. Public Health Nurs. 25 (3), 203–211. 10.1111/j.1525-1446.2008.00697.x 18477371

[B7] Ferguson W. J. Candib L. M. (2002). Culture, Language, and the Doctor-Patient Relationship. Fam. Med. 34 (5), 353–361. 12038717

[B8] Kales H. C. Gitlin L. N. Lyketsos C. G. (2015). Assessment and Management of Behavioral and Psychological Symptoms of Dementia. BMJ 350, h369. 10.1136/bmj.h369 25731881 PMC4707529

[B9] Kim H. M. Chiang C. Kales H. C. (2011). After the Black Box Warning: Predictors of Psychotropic Treatment Choices for Older Patients with Dementia. Psychiatr. Serv. 62 (10), 1207–1214. 10.1176/ps.62.10.pss6210_1207 21969648

[B10] Kirkham J. Sherman C. Velkers C. Maxwell C. Gill S. Rochon P. (2017). Antipsychotic Use in Dementia. Can. J. Psychiatry 62 (3), 170–181. 10.1177/0706743716673321 28212496 PMC5317021

[B11] Liperoti R. Pedone C. Corsonello A. (2008). Antipsychotics for the Treatment of Behavioral and Psychological Symptoms of Dementia (BPSD). Curr. Neuropharmacol 6 (2), 117–124. 10.2174/157015908784533860 19305792 PMC2647149

[B12] Maust D. T. Kim H. M. Seyfried L. S. Chiang C. Kavanagh J. Schneider L. S. (2015). Antipsychotics, Other Psychotropics, and the Risk of Death in Patients with Dementia: Number Needed to Harm. JAMA Psychiatry 72 (5), 438–445. 10.1001/jamapsychiatry.2014.3018 25786075 PMC4439579

[B13] Medicare Current Beneficiary Survey (MCBS) (2019). Medicare Current Beneficiary Survey (MCBS) | CMS Published December 6, 2019. Centers for Medicare & Medicaid Services (online). Available at: https://www.cms.gov/Research-Statistics-Data-and-Systems/Research/MCBS (Accessed April 24, 2020).

[B14] Raivio M. M. Laurila J. V. Strandberg T. E. Tilvis R. S. Pitkälä K. H. (2007). Neither atypical Nor Conventional Antipsychotics Increase Mortality or Hospital Admissions Among Elderly Patients with Dementia: a Two-Year Prospective Study. Am. J. Geriatr. Psychiatry 15 (5), 416–424. 10.1097/JGP.0b013e31802d0b00 17463191

[B15] Reed C. Belger M. Dell'agnello G. Wimo A. Argimon J. M. Bruno G. (2014). Caregiver Burden in Alzheimer's Disease: Differential Associations in Adult-Child and Spousal Caregivers in the GERAS Observational Study. Dement Geriatr. Cogn. Dis. Extra 4 (1), 51–64. 10.1159/000358234 24711814 PMC3977221

[B16] Reus V. I. Fochtmann L. J. Eyler A. E. Hilty D. M. Horvitz-Lennon M. Jibson M. D. (2016). The American Psychiatric Association Practice Guideline on the Use of Antipsychotics to Treat Agitation or Psychosis in Patients with Dementia. Am. J. Psychiatry 173 (5), 543–546. 10.1176/appi.ajp.2015.173501 27133416

[B17] Rigby T. Ashwill R. T. Johnson D. K. Galvin J. E. (2019). Differences in the Experience of Caregiving between Spouse and Adult Child Caregivers in Dementia with Lewy Bodies. Innov. Aging 3 (3), igz027. 10.1093/geroni/igz027 31528714 PMC6736163

[B18] Rochon P. A. Normand S. L. Gomes T. Gill S. S. Anderson G. M. Melo M. (2008). Antipsychotic Therapy and Short-Term Serious Events in Older Adults with Dementia. Arch. Intern. Med. 168 (10), 1090–1096. 10.1001/archinte.168.10.1090 18504337

[B19] Rosenheck R. A. Leslie D. L. Sindelar J. L. Miller E. A. Tariot P. N. Dagerman K. S. (2007). Cost-benefit Analysis of Second-Generation Antipsychotics and Placebo in a Randomized Trial of the Treatment of Psychosis and Aggression in Alzheimer Disease. Arch. Gen. Psychiatry 64 (11), 1259–1268. 10.1001/archpsyc.64.11.1259 17984395

[B20] Sepassi A. Watanabe J. H. (2019). Emergency Department Visits for Psychotropic-Related Adverse Drug Events in Older Adults with Alzheimer Disease, 2013-2014. Ann. Pharmacother. 53 (12), 1173–1183. 10.1177/1060028019866927 31342766

[B21] Tampi R. R. Tampi D. J. Balachandran S. Srinivasan S. (2016). Antipsychotic Use in Dementia: A Systematic Review of Benefits and Risks from Meta-Analyses. Ther. Adv. Chronic Dis. 7 (5), 229–245. 10.1177/2040622316658463 27583123 PMC4994396

[B22] van der Flier W. M. Scheltens P. (2005). Epidemiology and Risk Factors of Dementia. J. Neurol. Neurosurg. Psychiatry 76 (Suppl. 5), v2–7. 10.1136/jnnp.2005.082867 16291918 PMC1765715

[B23] Xiong G. L. Filshtein T. Beckett L. A. Hinton L. (2015). Antipsychotic Use in a Diverse Population with Dementia: A Retrospective Review of the National Alzheimer's Coordinating Center Database. J. Neuropsychiatry Clin. Neurosci. 27 (4), 326–332. 10.1176/appi.neuropsych.15010020 26488486 PMC4617662

[B24] Zhai Y. Yin S. Zhang D. (2016). Association between Antipsychotic Drugs and Mortality in Older Persons with Alzheimer's Disease: A Systematic Review and Meta-Analysis. J. Alzheimers Dis. 52 (2), 631–639. 10.3233/JAD-151207 27031490

